# Sophocarpine Alleviates Renal Ischemia–Reperfusion Injury by Mitigating Oxidative Stress and Mitochondrial Dysfunction via the SIRT1/PGC-1α Axis

**DOI:** 10.3390/biomedicines14061357

**Published:** 2026-06-16

**Authors:** Zhan Chen, Qiangmin Qiu, Dalin He, Bo Yu, Nan Jiang, Yujie Zhou, Tianyu Wang, Jiefu Zhu, Tao Qiu, Jiangqiao Zhou

**Affiliations:** Department of Organ Transplantation, Renmin Hospital of Wuhan University, Wuhan 430060, China; applepure@163.com (Z.C.); 2015302180359@whu.edu.cn (Q.Q.); 17326375859@163.com (D.H.); yubo1995@whu.edu.cn (B.Y.); zhouyujie0514@163.com (Y.Z.);

**Keywords:** sophocarpine, renal ischemia–reperfusion injury, acute kidney injury, oxidative stress, mitochondrial dysfunction, SIRT1, PGC-1α

## Abstract

**Background/Objectives**: Renal ischemia–reperfusion injury (IRI) is a major cause of acute kidney injury and delayed graft function after kidney transplantation. Oxidative stress, mitochondrial dysfunction, and tubular epithelial cell apoptosis are central events in renal IRI. Sophocarpine (SOP), a quinolizidine alkaloid derived from Sophora species, has reported antioxidant and anti-apoptotic activities, but its effects in renal IRI remain unclear. This study investigated the role and function of SOP in renal IRI. **Methods**: A bilateral renal IRI mouse model and a hypoxia/reoxygenation (H/R) model in HK-2 human proximal tubular epithelial cells were used. Renal function, histological injury, apoptosis, reactive oxygen species, malondialdehyde, superoxide dismutase activity, glutathione, mitochondrial morphology, mitochondrial membrane potential, and mitochondrial dynamics-related proteins were evaluated. SIRT1 dependency was examined using Sirt1 small interfering RNA in HK-2 cells and EX527-mediated SIRT1 inhibition in mice. **Results**: SOP pretreatment reduced serum creatinine and blood urea nitrogen levels, attenuated tubular injury and apoptosis, decreased oxidative stress, and preserved mitochondrial morphology and function after renal IRI. Similar protective effects were observed in HK-2 cells exposed to H/R. SOP increased SIRT1 and PGC-1α expression, whereas Sirt1 knockdown or pharmacological SIRT1 inhibition weakened the antioxidant and mitochondrial protective effects of SOP. **Conclusions**: SOP attenuates renal IRI-associated oxidative stress and mitochondrial dysfunction, at least in part through the SIRT1/PGC-1α axis. These findings support further investigation of SOP as a candidate renoprotective compound for ischemic kidney injury.

## 1. Introduction

Renal ischemia–reperfusion injury (IRI) refers to tissue injury that develops when renal blood flow is restored after a period of ischemia. It is common during major surgery and is particularly relevant to kidney transplantation, where it contributes to delayed graft function and may compromise long-term graft survival [1,2,3]. The pathogenesis of renal IRI is multifactorial and involves endothelial dysfunction, inflammation, tubular epithelial cell injury, oxidative stress, mitochondrial damage, and regulated cell death [4,5,6]. Among these processes, oxidative stress is especially important during reperfusion; the abrupt reintroduction of oxygen drives excessive reactive oxygen species (ROS) production, while endogenous antioxidant systems such as glutathione (GSH) and superoxide dismutase (SOD) can be consumed or functionally impaired. The resulting redox imbalance promotes lipid peroxidation, protein damage, DNA injury, inflammation, and apoptosis [4,5,6,7].

Mitochondria are both major sources and major targets of ROS during IRI [8]. Ischemia compromises oxidative phosphorylation and ionic homeostasis, whereas reperfusion accelerates electron leakage, ROS generation, mitochondrial permeability transition, and loss of mitochondrial membrane potential (MMP). Mitochondrial homeostasis is not limited to energy production; it also includes mitochondrial dynamics, biogenesis, mitophagy, membrane potential maintenance, and quality control. Disturbance of these processes contributes to tubular epithelial cell dysfunction and death. Experimental studies indicate that ROS scavenging and mitochondrial protection can improve renal function after IRI [9,10]. Sirtuin 1 (SIRT1), a NAD+-dependent deacetylase, regulates mitochondrial metabolism, antioxidant responses, and cellular stress resistance. Peroxisome proliferator-activated receptor gamma coactivator 1-alpha (PGC-1α), a downstream metabolic regulator of SIRT1, is involved in mitochondrial biogenesis and oxidative metabolism. Previous studies have linked the SIRT1/PGC-1α axis to renal protection under ischemia–reperfusion conditions [11,12].

**Figure 1:**
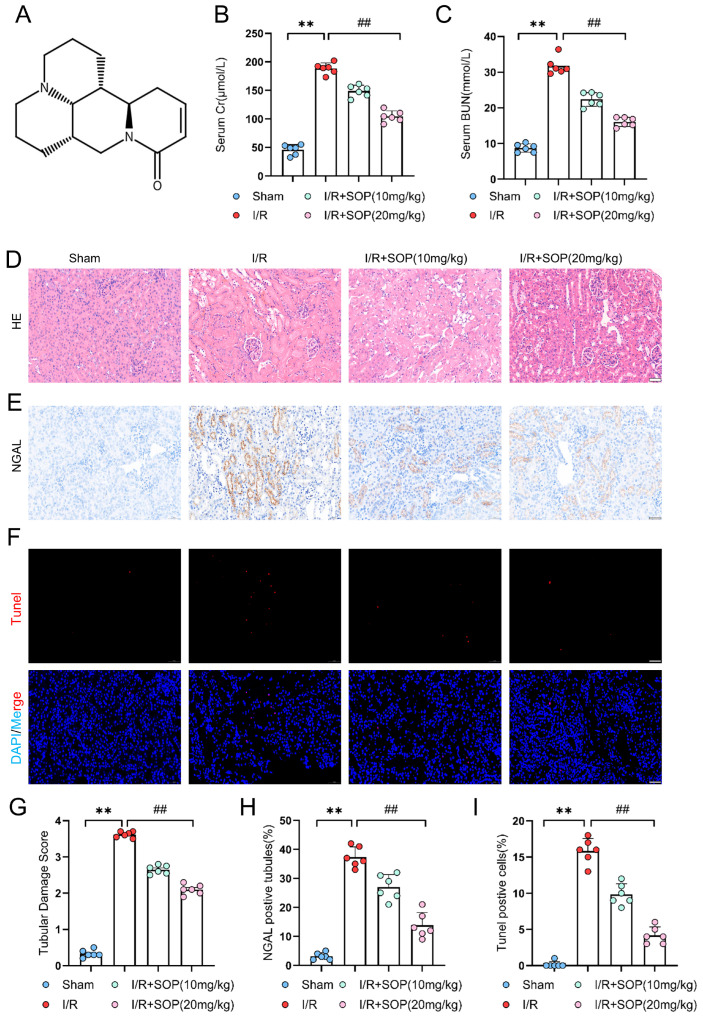
SOP protected the kidney from IRI in mice. (**A**) Chemical structure of SOP. (**B**,**C**) Serum Cr and BUN levels in mice from the indicated groups. (**D**,**G**) Representative H&E-stained renal sections and tubular injury scores. Scale bar = 50 μm. (**E**,**H**) Representative NGAL immunohistochemical staining and quantification. Scale bar = 50 μm. (**F**,**I**) Representative TUNEL staining and quantification of TUNEL-positive cells. Scale bar = 50 μm. Values are expressed as the mean ± SEM; *n* = 6. ** *p* < 0.01 vs. sham; ## *p* < 0.01 vs. I/R.

Traditional Chinese medicine-derived compounds have attracted attention as potential sources of therapies for acute kidney injury (AKI) [13,14]. Sophocarpine (SOP) is a quinolizidine alkaloid isolated from *Sophora alopecuroides*, *Sophora subprostrata*, and related Sophora species. The chemical structure of SOP is shown in Figure 1A. Published studies have reported anti-inflammatory, analgesic, antiviral, anticancer, antioxidant, and anti-apoptotic activities of SOP [15,16]. SOP has also been reported to regulate transient receptor potential ankyrin 1 (TRPA1) and transient receptor potential vanilloid 1 (TRPV1) channels [16]. In other injury models, SOP alleviated testicular IRI through the protein kinase B (AKT) pathway [17], reduced avermectin-induced kidney injury through the Nrf2/SLC7A11/GPX4 pathway [18], attenuated isoproterenol-induced renal injury [19], and suppressed inflammatory signaling in lupus nephritis [20]. However, whether SOP protects against renal IRI and how it affects mitochondrial homeostasis in this setting remain incompletely understood.

In this study, we used a murine renal IRI model and an H/R model in HK-2 human proximal tubular epithelial cells to evaluate the renoprotective effects of SOP. We organized the experimental workflow around three questions: whether SOP improves renal injury and cell survival, whether SOP mitigates oxidative stress and mitochondrial dysfunction, and whether SIRT1/PGC-1α signaling contributes to these effects. To address these questions, we assessed renal function, histological injury, apoptosis, oxidative stress markers, mitochondrial morphology, MMP, mitochondrial dynamics-related proteins, and SIRT1/PGC-1α signaling in vivo and in vitro.

## 2. Materials and Methods

### 2.1. Experimental Animals, Renal IRI Model, and In Vivo Drug Administration

C57BL/6 male mice (6–8 weeks old, 20–25 g) were obtained from the Experimental Animal Center of Wuhan University and maintained under specific pathogen-free conditions. Mice were randomly allocated to the Sham, I/R, I/R + SOP 10 mg/kg, I/R + SOP 20 mg/kg, and I/R + SOP 20 mg/kg + EX527 groups (*n* = 6 mice per group). A bilateral renal IRI model was established as described previously [21]. Briefly, both renal pedicles were exposed and occluded with non-traumatic vascular clamps for 30 min, followed by clamp removal to allow reperfusion for 24 h. In sham-operated mice, the renal pedicles were exposed but not clamped. 10 mM SOP in DMSO (MCE, Cat. No. HY-N0103, South Brunswick, NJ, USA) was dissolved in saline (5 mg/mL) and administered intraperitoneally once daily for 3 consecutive days before surgery at 10 or 20 mg/kg. Vehicle-treated mice received the same volume of DMSO-containing vehicle (5% DMSO in saline,10 mL/kg). The 10 and 20 mg/kg doses were selected based on published SOP studies and preliminary tolerability observations [15,17,19]. EX527, a selective SIRT1 inhibitor, was administered (10 mg/kg) in the mechanistic inhibition experiment. All procedures complied with the animal care guidelines of Wuhan University and Renmin Hospital of Wuhan University and were approved by the Experimental Animal Ethics Committee (Approval No. WDRM20250309B).

### 2.2. Renal Function Assessment and Sample Collection

At 24 h after reperfusion, mice were deeply anesthetized with 1% pentobarbital sodium (0.1 mL, 50 mg/kg). Blood samples were collected by retro-orbital venous puncture at the terminal endpoint under deep anesthesia. Mice were then euthanized by cervical dislocation, and kidneys were harvested for histology, immunostaining, biochemical assays, electron microscopy, RNA extraction, and protein extraction. Serum was separated after clotting and centrifugation. Serum creatinine (Cr) and blood urea nitrogen (BUN) were measured using commercial kits from Nanjing Jiancheng Bioengineering Institute (C011-2-1 and C013-2-1, respectively), according to the manufacturer’s instructions.

### 2.3. Oxidative Stress Assays

Renal cortical tissue and HK-2 cells were homogenized or lysed on ice in PBS buffer. After centrifugation, supernatants were collected for oxidative stress assays. Malondialdehyde (MDA) concentration was quantified using the Lipid Peroxidation MDA Assay Kit (Beyotime, Cat. No. S0131S, Shanghai, China), with absorbance recorded at 532 nm. Total glutathione (GSH) content was assessed using the Total Glutathione Assay Kit (Beyotime, Cat. No. S0052, Shanghai, China), with absorbance recorded at 412 nm. SOD enzymatic activity was measured using the Total Superoxide Dismutase Assay Kit (Beyotime, Cat. No. S0101S, Shanghai, China), with absorbance recorded at 450 nm. Protein concentration was determined by the bicinchoninic acid (BCA) assay (Beyotime, Cat. No. P0010S, Shanghai, China). Data were normalized to protein concentration and expressed relative to the corresponding control group. These assays were used as selected indicators of lipid peroxidation, enzymatic antioxidant capacity, and non-enzymatic antioxidant reserve.

### 2.4. HK-2 Cell Culture, H/R Model, SOP Treatment, and Sirt1 Knockdown

HK-2 human proximal tubular epithelial cells were obtained from China Center for Type Culture Collection and cultured in complete DMEM/F12 medium at 37 °C in a humidified incubator containing 5% CO_2_. To establish the in vitro ischemia–reperfusion model, cells were subjected to hypoxia/reoxygenation (H/R): 12 h in serum-free DMEM/F12 medium under 1% O_2_ at 37 °C, followed by 6 h of reoxygenation in complete medium under normoxic conditions [22]. SOP was administered 24 h before H/R at 5, 10, 20, 40, 80, 160, or 200 μM for dose–response testing; 20 and 80 μM were used for subsequent mechanistic experiments. DMSO was used as the solvent control at the same final concentration in all groups (1% DMSO in PBS). For gene silencing, Sirt1 small interfering RNA (si-Sirt1) and negative-control siRNA (si-NC) were obtained from Sangon Biotech (Shanghai, China). Transfection was performed using Lipofectamine 3000 (Thermo Fisher Scientific, Cat. No. L3000001, Shanghai, China) according to the manufacturer’s protocol. The si-Sirt1 sequences were: sense, 5′-CCCUCAAAGUAAGACCAGUTT-3′; antisense, 5′-ACUGGUCUUACUUUGAGGGAA-3′.

### 2.5. Histology, Immunohistochemistry, and Transmission Electron Microscopy

Kidney samples for routine histology were fixed in 4% paraformaldehyde, embedded in paraffin, sectioned at 4 μm, and stained with hematoxylin and eosin (H&E). Tubular injury was assessed based on brush border loss, tubular dilation, cast formation, and epithelial cell lysis. Two senior renal pathologists blinded to group allocation scored renal injury according to the percentage of damaged tubules: 0, normal; 1, <25%; 2, 25–50%; 3, 50–75%; and 4, >75%. Quantification was performed in five randomly selected cortical fields per mouse (*n* = 6 mice per group). For transmission electron microscopy (TEM), kidney tissues were fixed with electron microscopy fixative (Servicebio, Cat. No. G1102, Wuhan, China) at temperature and duration, post-fixed, dehydrated, embedded, sectioned, stained, and examined by TEM. Mitochondrial damage was defined by swelling, loss of electron density, cristae disruption, vacuolization, or fragmentation and was quantified as 20% percentage of damaged mitochondrial area. For immunohistochemistry (IHC), 4 μm paraffin sections were deparaffinized, rehydrated, subjected to microwave-assisted antigen retrieval in citrate buffer, treated with 3% H_2_O_2_, blocked with 3% bovine serum albumin for 30 min, and incubated overnight at 4 °C with anti-NGAL (Abclonal, Cat. No. A26627PM, 1:300, Wuhan, China) or anti-SIRT1 (Huabio, Cat. No. ER130811; 1:200, Hangzhou, China). After incubation with HRP-conjugated secondary antibody, (Invitrogen; Cat. No. C31460100; 1:1000, Shanghai, China), sections were developed with diaminobenzidine (DAB), counterstained with hematoxylin, and imaged at 200× magnification. Isotype and no-primary-antibody controls were included. IHC staining was quantified using ImageJ v1.54g (ImageJ, NIH, Bethesda, ML, USA) as positive staining area in randomly selected cortical fields.

### 2.6. TdT-Mediated dUTP Nick-End Labeling (TUNEL)

TUNEL staining was performed on 4 μm paraffin-embedded kidney sections using the Beyotime C1090 kit (Shanghai, China) according to the manufacturer’s instructions. After permeabilization and labeling, sections were washed, counterstained with DAPI, and examined using an Olympus IX51 fluorescence microscope (Olympus Corporation, Tokyo, Japan) [17]. At least five randomly selected cortical fields per section were analyzed, and apoptosis was quantified as the percentage of TUNEL-positive nuclei among total DAPI-positive nuclei.

### 2.7. Immunofluorescence and ROS Detection

For cell immunofluorescence, HK-2 cells were seeded on culture plates or coverslips, washed with phosphate-buffered saline (PBS), fixed with 4% paraformaldehyde for 30 min at room temperature, permeabilized with 0.1% Triton X-100 for 10 min, and blocked with 10% Goat Serum. Cells were incubated overnight at 4 °C with primary antibodies against TOM20 (Proteintech, Cat. No. 11802-1-AP; 1:250, Wuhan, China) or SIRT1 (Huabio, Cat. No. ER130811; 1:300, Hangzhou, China), followed by fluorophore-conjugated secondary antibodies (Proteintech, Cat. No. SA00013-4; 1:1000, Wuhan, China). For in vivo ROS detection, fresh frozen kidney sections were incubated with dihydroethidium (DHE; Beyotime, Cat. No. S0063, Shanghai, China) according to the manufacturer’s instructions and imaged promptly to minimize signal decay. For in vitro ROS detection, HK-2 cells were incubated with DCFH-DA (Beyotime, Cat. No. S0033S, Shanghai, China). Fluorescence images were captured using an Olympus IX51 microscope (Olympus Corporation, Tokyo, Japan) under identical exposure settings within each experiment. Fluorescence intensity was quantified in randomly selected 10 fields of view per group using ImageJ v1.54g (ImageJ, NIH, USA) (*n* = 6).

### 2.8. Mitochondrial Membrane Potential Assay

Mitochondrial membrane potential (MMP) in HK-2 cells was assessed using a JC-1 staining kit (Beyotime, Cat. No. C2006, Shanghai, China). Cells were incubated with JC-1 working solution in the dark according to the manufacturer’s instructions, washed with assay buffer, and imaged with an Olympus IX51 fluorescence microscope (Olympus Corporation, Tokyo, Japan). MMP changes were evaluated by the red-to-green fluorescence ratio in 3 randomly selected fields per group (*n* = 6).

### 2.9. Real-Time Quantitative PCR

Total RNA was extracted from kidney tissue and HK-2 cells using TRIzol reagent (Solarbio, Cat. No. R1100, Beijing, China). RNA concentration and purity were measured by Nanodrop. Complementary DNA was synthesized using the SuperScript First-Strand Synthesis Kit (Invitrogen, Shanghai, China) according to the manufacturer’s protocol. Real-time quantitative PCR (RT-qPCR) was performed using qPCR master mix, instrument, cycling parameters. β-actin (ACTB) served as the internal control, and relative expression was calculated by the 2^−ΔΔCt^ method. Primer sequences for ACTB, SOD1, SOD2, Catalase, SIRT1, and PGC-1α was provided in Table 1.

### 2.10. Western Blotting

Mouse kidney tissue and HK-2 cells were lysed on ice using RIPA buffer (Biosharp, Cat. No. BL509A, Beijing, China) containing protease (Biosharp, BL612A) and phosphatase inhibitors (Biosharp, BL1439A). Lysates were centrifuged to remove insoluble debris, and protein concentration was determined using the BCA assay. Equal amounts of protein were separated on 10–12% SDS-polyacrylamide gels and transferred to nitrocellulose membranes (Cytiva, Cat. No. 10600001, Shanghai, China). Membranes were blocked with 5% skim milk for 1–2 h and incubated overnight at 4 °C with the primary antibodies listed in Table 2. After three washes with Tris-buffered saline containing Tween 20 (TBST), membranes were incubated with HRP-conjugated secondary antibodies (goat anti-rabbit IgG (Biosharp, Cat. No. BL003A, 1:10,000, Beijing, China)) and goat anti-mouse IgG (Biosharp, Cat. No. BL001A, 1:10,000, Beijing, China) for 1–2 h at room temperature. Signals were developed using chemiluminescent HRP substrate (Biosharp, Beijing, China) and quantified with ImageJ v1.54g software (NIH, USA). ACTB was used as the loading control.

### 2.11. Statistical Analysis

GraphPad Prism 10.0 (GraphPad Software, San Diego, CA, USA) was used for statistical analysis. Data are presented as the mean ± standard error of the mean (SEM). Each cell experiment was performed at least three independent times, and animal group sizes are indicated in the figure legends. For comparisons between two groups, an unpaired two-tailed Student’s *t*-test was used when normality and homogeneity of variance assumptions were met. For comparisons among multiple groups, one-way analysis of variance (ANOVA) followed by Tukey’s post hoc test was used. A *p*-value < 0.05 was considered statistically significant.

## 3. Results

### 3.1. SOP Pretreatment Attenuates Renal Dysfunction, Tubular Injury, and Apoptosis After Renal IRI

To determine whether SOP protects the kidney during renal IRI, mice were subjected to 30 min bilateral renal ischemia followed by 24 h of reperfusion. Compared with the sham group, I/R markedly increased serum Cr and BUN levels (Figure 1B,C). SOP pretreatment reduced both parameters, and the 20 mg/kg dose produced a stronger protective effect than the 10 mg/kg dose. H&E staining showed tubular dilation, brush border loss, cast formation, and epithelial cell lysis after I/R; these pathological changes were attenuated by SOP pretreatment (Figure 1D,G). Consistently, NGAL, a notable marker of tubular injury [23], immunohistochemical staining showed increased NGAL expression after I/R, whereas SOP pretreatment reduced NGAL staining in renal tissue (Figure 1E,H). TUNEL staining further showed that SOP reduced I/R-induced renal tubular apoptosis (Figure 1F,I). These results indicate that SOP pretreatment attenuates renal dysfunction, tubular injury, and apoptosis in the murine IRI model.

### 3.2. SOP Attenuates Oxidative Stress in Mouse Kidneys After I/R

**Figure 2:**
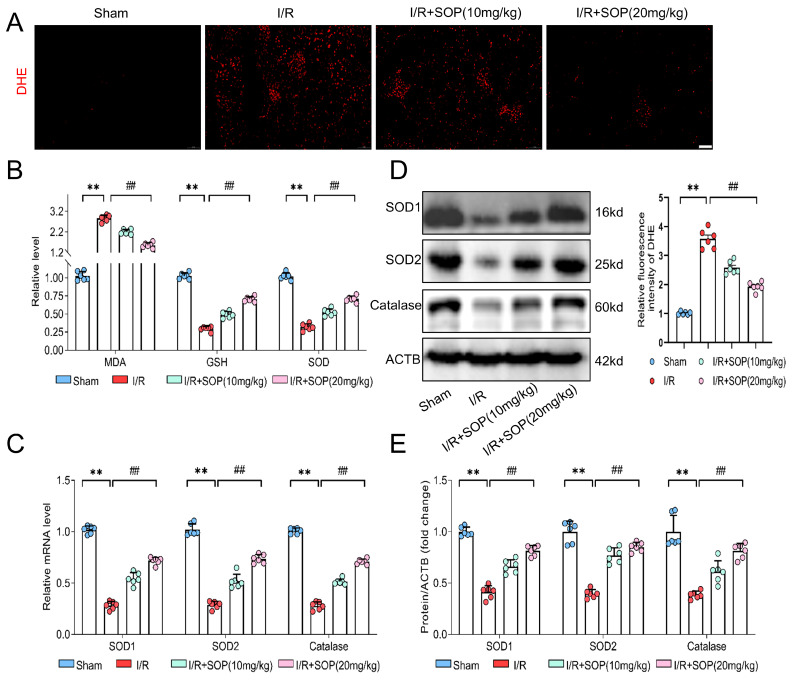
SOP alleviated IRI-induced renal oxidative stress. (**A**) Representative DHE staining images of mouse kidney sections. Scale bar = 50 μm. (**B**) Quantitative analysis of MDA, GSH, and SOD levels in kidney tissue. (**C**) RT-qPCR analysis of SOD1, SOD2, and Catalase mRNA expression. (**D**,**E**) Western blot analysis and quantification of SOD1, SOD2, and Catalase protein expression in mouse kidney tissue. Values are expressed as the mean ± SEM; *n* = 6. ** *p* < 0.01 vs. sham; ## *p* < 0.01 vs. I/R.

We next examined whether SOP affected renal oxidative stress after I/R. DHE staining showed a marked increase in ROS signal in I/R kidneys compared with sham kidneys, whereas SOP pretreatment reduced the DHE fluorescence signal (Figure 2A). Biochemical assays showed that I/R decreased GSH content and SOD activity and increased MDA accumulation; SOP pretreatment partially reversed these changes (Figure 2B). Consistent with the biochemical data, RT-qPCR and Western blot analyses showed that antioxidant enzymes, including Catalase, SOD1, and SOD2, were downregulated after I/R and restored by SOP pretreatment (Figure 2C–E). Together, these data show that SOP reduces selected oxidative stress endpoints and supports antioxidant defense after renal I/R.

### 3.3. SOP Preserves Mitochondrial Ultrastructure and Mitochondrial Dynamics in Mouse Kidneys After I/R

**Figure 3:**
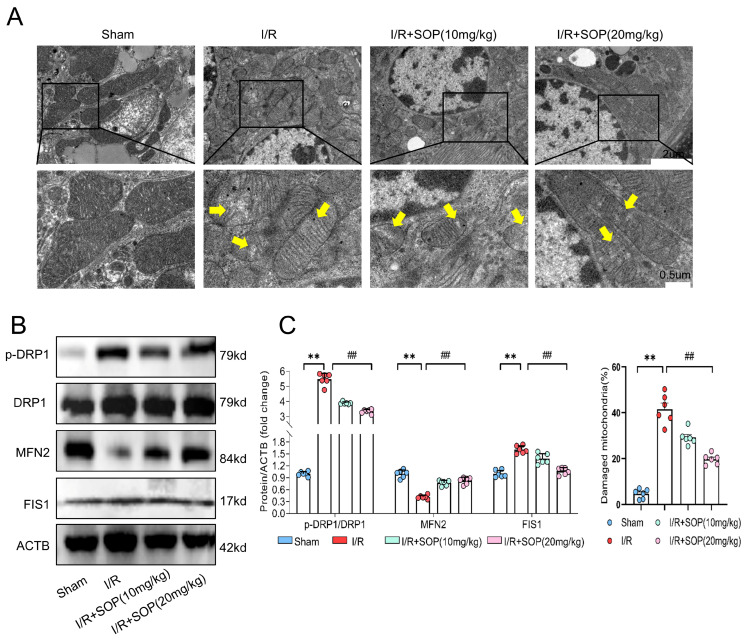
SOP attenuated IRI-induced mitochondrial injury in mouse kidneys. (**A**) Representative TEM images of renal tubular mitochondria. Yellow arrows in the TEM panels indicate areas of mitochondrial damage. Scale bar = 2 μm (**upper panels**) and 0.5 μm (**lower panels**). (**B**,**C**) Western blot analysis and quantification of FIS1, p-DRP1 (Ser616), DRP1, and MFN2 expression in kidney tissue. Values are expressed as the mean ± SEM; *n* = 6. ** *p* < 0.01 vs. sham; ## *p* < 0.01 vs. I/R.

Because mitochondrial injury is a key contributor to renal IRI, mitochondrial ultrastructure and dynamics-related proteins were evaluated. TEM analysis showed that I/R caused mitochondrial swelling, fragmentation, cristae disruption, and vacuole-like structural changes in renal tubular cells, whereas SOP pretreatment alleviated these ultrastructural abnormalities (Figure 3A). Western blot analysis further showed that I/R increased the fission-related proteins FIS1 and phosphorylated DRP1 at Ser616 and reduced the fusion-related protein MFN2 (Figure 3B,C). SOP pretreatment reduced FIS1 and p-DRP1 (Ser616) and restored MFN2 expression. These results suggest that SOP helps preserve mitochondrial integrity and modulates mitochondrial dynamics after renal I/R.

### 3.4. SOP Attenuates H/R-Induced Oxidative Stress in HK-2 Cells

**Figure 4:**
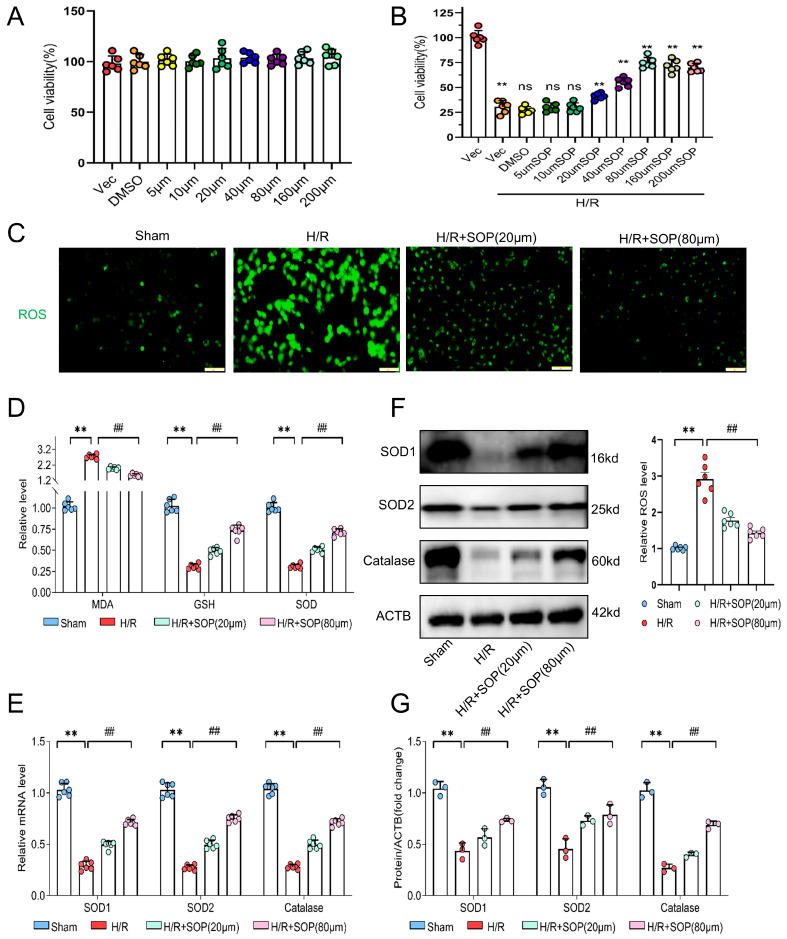
SOP attenuated oxidative stress during H/R in HK-2 cells. (**A**,**B**) CCK-8 assay results showing cell viability under the indicated treatment conditions. (**C**) Representative DCFH-DA ROS staining images. Scale bar = 50 μm. (**D**) GSH, SOD, and MDA levels in HK-2 cells. (**E**) RT-qPCR analysis of SOD1, SOD2, and Catalase mRNA expression. (**F**,**G**) Western blot analysis and quantification of SOD1, SOD2, and Catalase protein expression. Values are expressed as the mean ± SEM; *n* = 3. ** *p* < 0.01 vs. control; ns, not significant; ## *p* < 0.01 vs. H/R.

To test the protective effect of SOP in vitro, HK-2 cells were exposed to H/R. SOP treatment under normoxic conditions did not significantly affect HK-2 cell viability (Figure 4A). After H/R injury, SOP pretreatment improved cell survival in a concentration-dependent manner, with 80 μM showing the strongest protective effect among the tested concentrations (Figure 4B). Therefore, 20 μM and 80 μM were selected for subsequent experiments. DCFH-DA staining showed that H/R increased intracellular ROS, whereas SOP pretreatment reduced ROS accumulation (Figure 4C). H/R also decreased GSH content and SOD activity and increased MDA levels; SOP pretreatment partially reversed these changes (Figure 4D). In addition, SOP restored the mRNA and protein expression of SOD1, SOD2, and Catalase that had been reduced by H/R (Figure 4E–G). These findings indicate that SOP attenuates H/R-induced oxidative stress in HK-2 cells.

### 3.5. SOP Improves H/R-Induced Mitochondrial Dysfunction in HK-2 Cells

**Figure 5:**
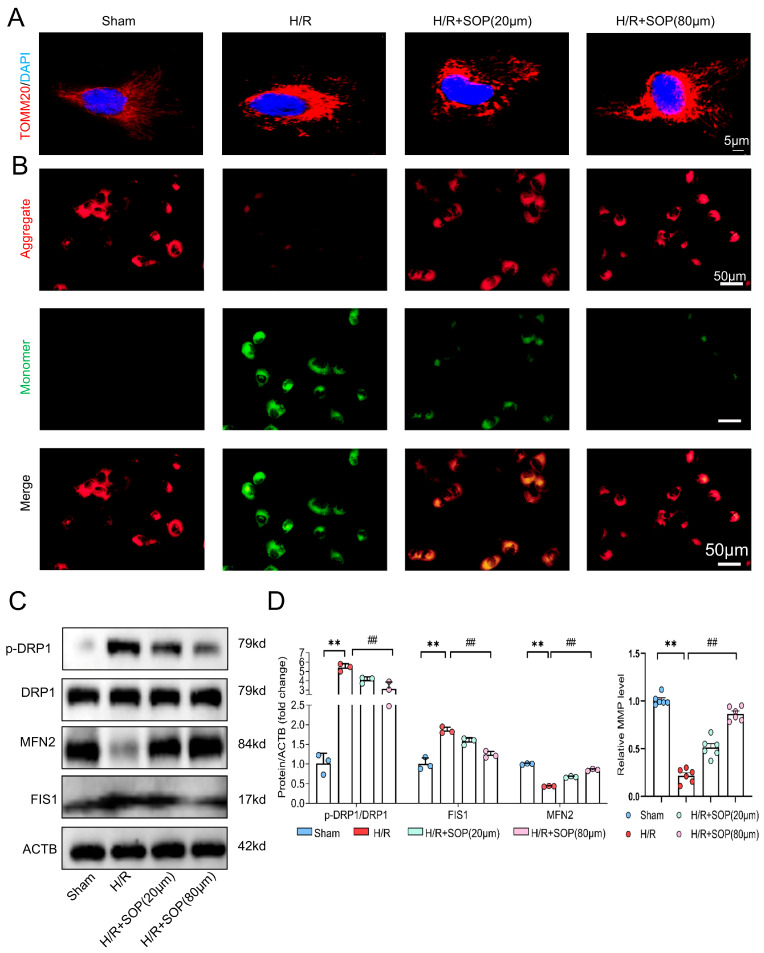
SOP preserved mitochondrial morphology and function in HK-2 cells following H/R. (**A**) Representative TOM20 immunofluorescence images. Scale bar = 5 μm. (**B**) Representative JC-1 images showing mitochondrial membrane potential. Scale bar = 50 μm. (**C**,**D**) Western blot analysis and quantification of p-DRP1 (Ser616)/DRP1, FIS1, and MFN2 protein expression. Values are expressed as the mean ± SEM; *n* = 3. ** *p* < 0.01 vs. control; ## *p* < 0.01 vs. H/R.

Mitochondrial morphology and function were then examined in HK-2 cells. TOM20 immunofluorescence showed elongated and interconnected mitochondria in control cells, whereas H/R exposure caused fragmented and punctate mitochondrial morphology (Figure 5A). SOP pretreatment partially improved mitochondrial morphology, with a more evident effect at 80 μM than at 20 μM. JC-1 staining showed that H/R reduced MMP, and SOP pretreatment partially restored the red-to-green fluorescence ratio (Figure 5B). Consistent with the in vivo findings, H/R increased FIS1 and p-DRP1 (Ser616) expression and decreased MFN2 expression, whereas SOP pretreatment attenuated these changes (Figure 5C,D). These results indicate that SOP mitigates H/R-induced mitochondrial dysfunction in HK-2 cells.

### 3.6. SOP Regulates the SIRT1/PGC-1α Signaling Axis In Vivo and In Vitro

**Figure 6:**
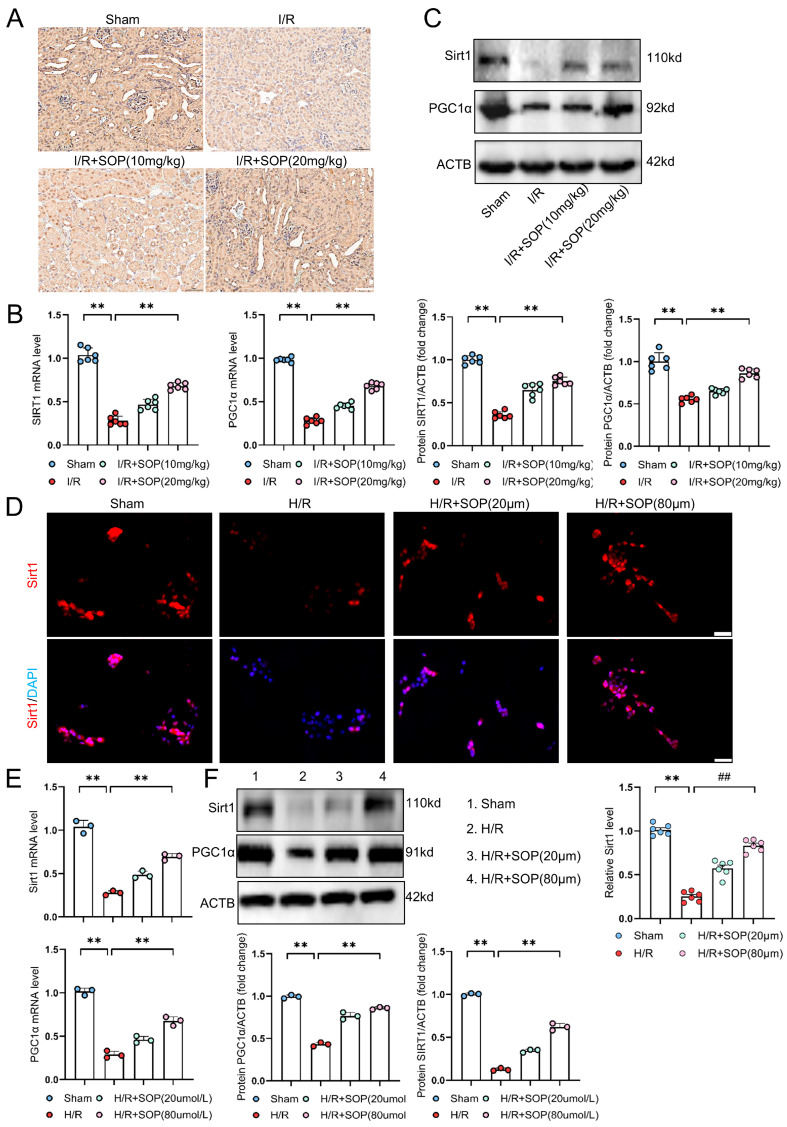
SOP upregulated SIRT1/PGC-1α signaling following IRI and H/R treatments. (**A**) Representative SIRT1 immunohistochemical staining in mouse kidney tissue. Scale bar = 50 μm. (**B**) RT-qPCR analysis of SIRT1 and PGC-1α mRNA expression in mouse kidney tissue. (**C**) Western blot analysis and quantification of SIRT1 and PGC-1α protein expression in mouse kidney tissue. (**D**) Representative SIRT1 immunofluorescence images in HK-2 cells. Scale bar = 50 μm. (**E**) RT-qPCR analysis of SIRT1 and PGC-1α mRNA expression in HK-2 cells. (**F**) Western blot analysis and quantification of SIRT1 and PGC-1α protein expression in HK-2 cells. Values are expressed as the mean ± SEM; *n* = 6 for mice and *n* = 3 for HK-2 cells. ** *p* < 0.01 vs. sham or control; ## *p* < 0.01 vs. I/R or H/R.

To explore the signaling mechanism associated with SOP-mediated protection, we examined the SIRT1/PGC-1α axis. IHC staining showed reduced SIRT1 expression in renal tissue after I/R, whereas SOP pretreatment partially restored SIRT1 staining (Figure 6A). RT-qPCR and Western blot analyses showed that SOP increased the expression of SIRT1 and its downstream regulator PGC-1α in mouse kidneys (Figure 6B,C). In HK-2 cells, H/R reduced SIRT1 immunofluorescence, and SOP pretreatment increased SIRT1 labeling (Figure 6D). RT-qPCR and Western blot analyses confirmed increased SIRT1 and PGC-1α expression after SOP pretreatment in the H/R model (Figure 6E,F). These results suggest that SOP is associated with activation of SIRT1/PGC-1α signaling under I/R and H/R conditions.

### 3.7. Sirt1 Knockdown Reduces SOP-Mediated Antioxidant and Mitochondrial Protection In Vitro

**Figure 7:**
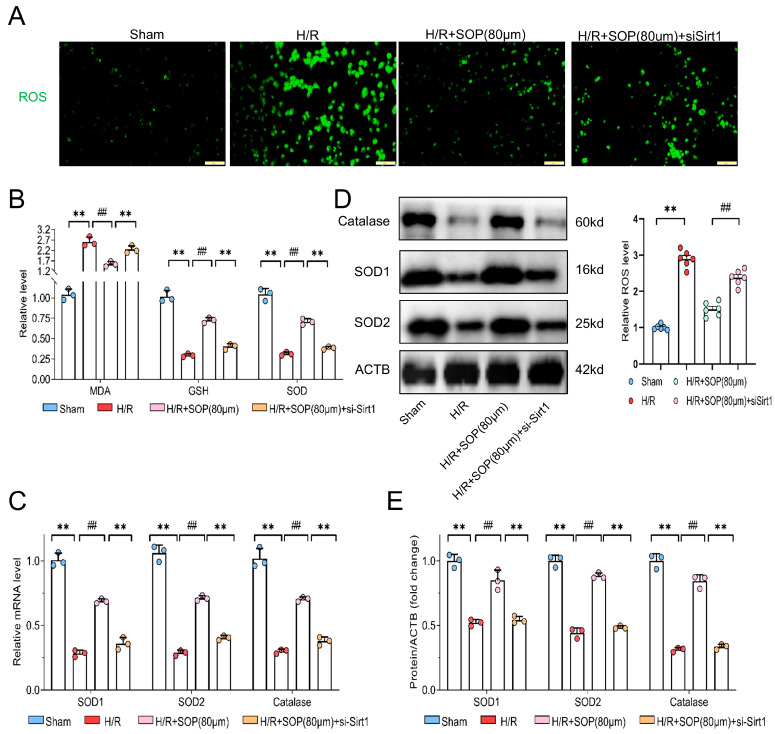
Sirt1 knockdown weakened the inhibitory effect of SOP on oxidative stress in HK-2 cells. (**A**) Representative DCFH-DA staining images. Scale bar = 50 μm. (**B**) GSH, SOD, and MDA levels in HK-2 cells under the indicated treatment conditions. (**C**) RT-qPCR analysis of SOD1, SOD2, and Catalase mRNA expression. (**D**,**E**) Western blot analysis and quantification of SOD1, SOD2, and Catalase protein expression. Values are expressed as the mean ± SEM; *n* = 3. ** *p* < 0.01 vs. control; ## *p* < 0.01 vs. H/R.

**Figure 8:**
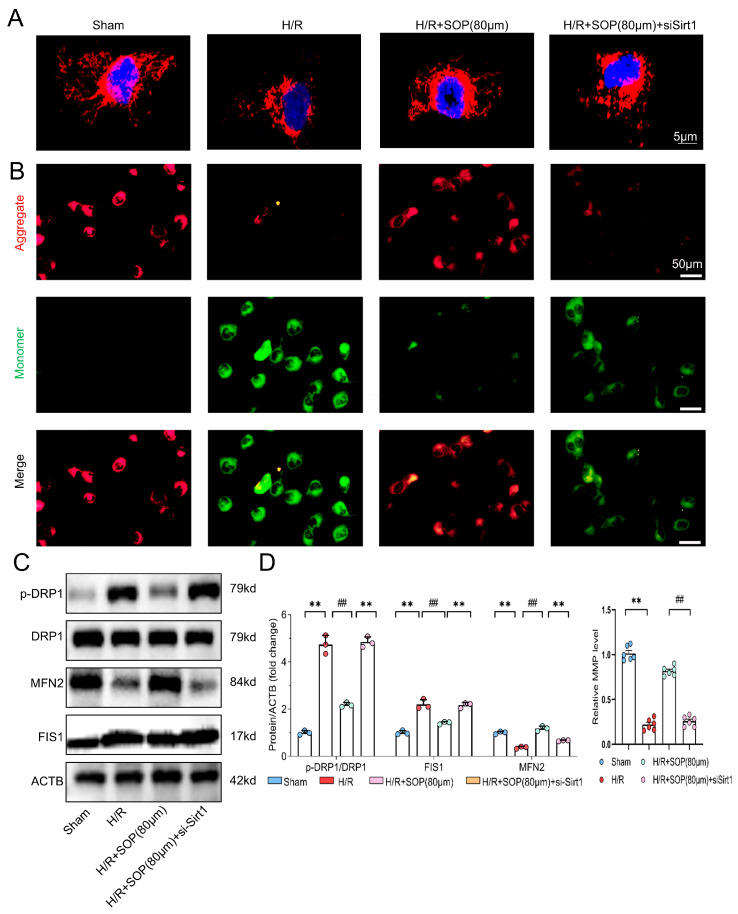
Sirt1 knockdown reduced the protective effect of SOP on mitochondria in HK-2 cells. (**A**) Representative TOM20 immunofluorescence images. Scale bar = 5 μm. (**B**) Representative JC-1 images showing mitochondrial membrane potential. Scale bar = 50 μm. (**C**,**D**) Western blot analysis and quantification of p-DRP1 (Ser616)/DRP1, FIS1, and MFN2 protein expression. Values are expressed as the mean ± SEM; *n* = 3. ** *p* < 0.01 vs. control; ## *p* < 0.01 vs. H/R.

To determine whether SIRT1 contributes to SOP-mediated cytoprotection, Sirt1 expression was silenced in HK-2 cells using siRNA. SOP reduced H/R-induced ROS accumulation, but this effect was weakened by Sirt1 knockdown (Figure 7A). Sirt1 knockdown also reduced the ability of SOP to restore GSH content and SOD activity and to decrease MDA accumulation (Figure 7B). In parallel, Sirt1 knockdown attenuated SOP-mediated restoration of SOD1, SOD2, and Catalase expression (Figure 7C–E). The mitochondrial protective effects of SOP were also reduced after Sirt1 knockdown: TOM20 staining showed persistent mitochondrial fragmentation, JC-1 staining showed impaired MMP recovery, and Western blot analysis showed reversal of SOP-mediated regulation of FIS1, p-DRP1 (Ser616), and MFN2 (Figure 8A–D). These findings indicate that SIRT1 is required, at least in part, for the antioxidant and mitochondrial protective effects of SOP in HK-2 cells.

### 3.8. Pharmacological SIRT1 Inhibition Reduces SOP-Mediated Renoprotection In Vivo

**Figure 9:**
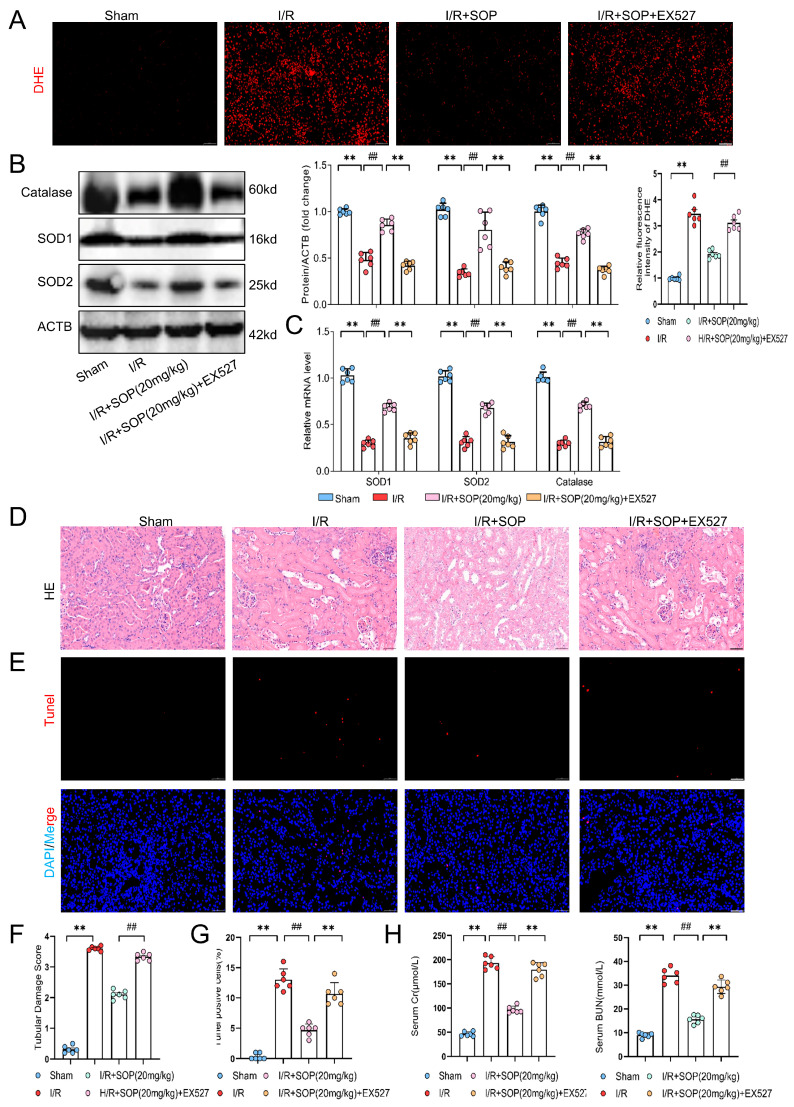
Pharmacological SIRT1 inhibition reduced the protective effect of SOP against renal IRI in vivo. (**A**) Representative DHE staining images of mouse kidney sections. Scale bar = 50 μm. (**B**,**C**) Western blot and RT-qPCR analyses of SOD1, SOD2, and Catalase expression in mouse kidney tissue. (**D**,**F**) Representative H&E-stained renal sections and tubular injury scores. Scale bar = 50 μm. (**E**,**G**) Representative TUNEL staining and quantification of TUNEL-positive cells. Scale bar = 50 μm. (**H**) Serum Cr and BUN levels in the indicated groups. Values are expressed as the mean ± SEM; *n* = 6. ** *p* < 0.01 vs. Sham; ## *p* < 0.01 vs. I/R.

To assess SIRT1 dependency in vivo, mice subjected to renal IRI were treated with SOP in the presence or absence of EX527, a selective SIRT1 inhibitor. Compared with SOP treatment alone, co-administration of EX527 increased renal ROS levels, as shown by DHE staining (Figure 9A). EX527 also blunted the SOP-mediated restoration of antioxidant defenses and was associated with lower GSH, SOD, and Catalase levels and higher MDA accumulation (Figure 9B,C). H&E and TUNEL staining showed more severe tubular injury and apoptosis in the SOP + EX527 group than in the SOP group (Figure 9D–G). Serum Cr and BUN levels were also higher after EX527 co-administration (Figure 9H). These data indicate that pharmacological SIRT1 inhibition weakens the protective effects of SOP against renal IRI in mice.

## 4. Discussion

**Figure 10:**
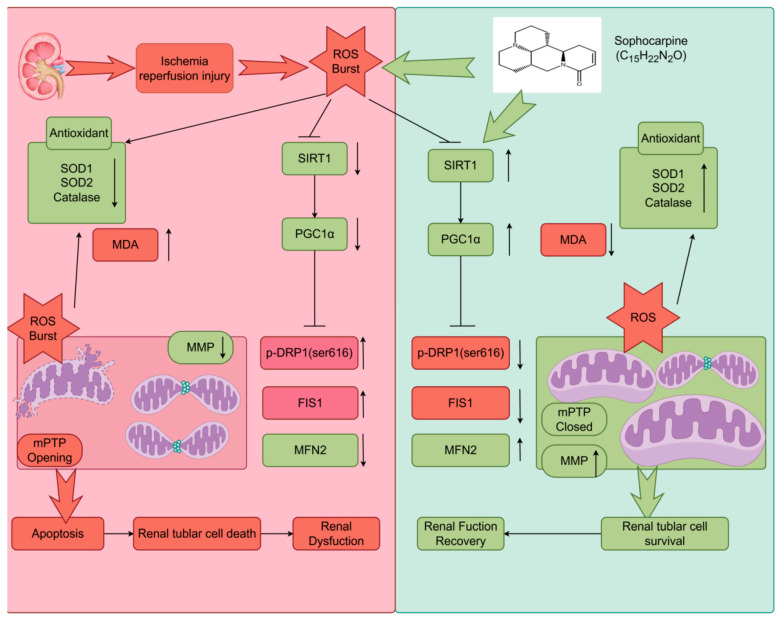
Proposed mechanism by which SOP alleviates renal ischemia–reperfusion injury by maintaining mitochondrial homeostasis and suppressing oxidative stress, with its efficacy partially dependent on activation of the SIRT1/PGC-1α pathway (A standard arrow (→) indicates activation. An upward arrow (↑) indicates an increased expression level (upregulation). A downward arrow (↓) indicates a decreased expression level (downregulation). A T-shaped arrow (—|) indicates inhibition).

This study demonstrates that SOP attenuates renal dysfunction, tubular epithelial cell injury, oxidative stress, apoptosis, and mitochondrial dysfunction in experimental renal IRI. In HK-2 cells exposed to H/R, SOP similarly reduced oxidative stress and preserved mitochondrial morphology and MMP. Mechanistically, SOP increased SIRT1 and PGC-1α expression, and Sirt1 knockdown or EX527-mediated SIRT1 inhibition weakened its protective effects. These findings support a model in which SOP protects renal tubular cells from I/R- or H/R-induced injury partly through the SIRT1/PGC-1α axis (Figure 10).

Renal IRI involves a rapid shift from ischemic metabolic stress to reperfusion-associated oxidative injury. Excessive ROS production can consume antioxidant reserves, impair antioxidant enzyme activity, and drive lipid peroxidation. In the present study, I/R and H/R increased ROS and MDA while reducing GSH and SOD activity, and SOP partially reversed these changes. We also observed restoration of SOD1, SOD2, and Catalase expression after SOP treatment. These findings are consistent with previous reports that SOP has antioxidant and anti-apoptotic effects in other injury models [17,18,19,20]. However, the redox assessment in this study was based on selected markers rather than comprehensive redox metabolomics or direct measurement of specific ROS. Therefore, our data support an antioxidant effect of SOP but do not fully define the complete redox metabolic state after I/R.

Mitochondrial damage is closely linked to oxidative stress in renal IRI. Mitochondria generate ROS during reperfusion, and ROS in turn disrupt mitochondrial membranes, cristae structure, and mitochondrial quality control [24,25,26,27]. Mitochondrial dynamics are essential for maintaining mitochondrial homeostasis: excessive fission can produce small dysfunctional mitochondria, whereas fusion supports mitochondrial networking and functional recovery [24,28,29,30,31]. In this study, I/R and H/R increased FIS1 and p-DRP1 (Ser616) and decreased MFN2, indicating a shift toward mitochondrial fission. SOP treatment reduced these fission-related changes and improved mitochondrial morphology and MMP. These findings suggest that SOP protects mitochondrial structure and function at least partly by modulating mitochondrial dynamics. The present data are also consistent with previous studies showing that inhibition of DRP1-FIS1-mediated fragmentation can attenuate acute kidney injury after ischemic stress [29,32,33].

The SIRT1/PGC-1α axis provides a plausible link between SOP treatment, antioxidant defense, and mitochondrial preservation. SIRT1 is a NAD+-dependent deacetylase that regulates stress responses, mitochondrial metabolism, and antioxidant pathways, while PGC-1α is a key regulator of mitochondrial biogenesis and oxidative metabolism [11,12]. Our data show that SOP increased SIRT1 and PGC-1α expression in both mouse kidneys and HK-2 cells, and loss of SIRT1 signaling by siRNA or EX527 reduced the protective effects of SOP. These findings support the involvement of SIRT1/PGC-1α signaling. Nevertheless, the upstream mechanism by which SOP increases SIRT1 remains unresolved. SOP may directly affect SIRT1 expression or activity, or it may act indirectly through changes in cellular NAD+/NADH balance, inflammatory signaling, oxidative burden, or other metabolic pathways. Future studies should measure SIRT1 enzymatic activity, NAD+ metabolism, and upstream regulatory pathways to clarify whether SOP is a direct SIRT1 activator or an indirect modulator.

This study has several limitations. First, SOP was administered before ischemic injury; therefore, the current design primarily supports a prophylactic effect and is most relevant to settings in which ischemia can be anticipated, such as transplantation. A therapeutic post-reperfusion dosing model is needed before broader clinical translation can be inferred. Second, intraperitoneal administration was used to ensure controlled systemic exposure in mice, but this route is not directly applicable to human use; oral or intravenous administration, pharmacokinetics, pharmacodynamics, bioavailability, and potential gastrointestinal metabolism require further study. Third, an SOP-alone in vivo group was not included, which limits evaluation of whether SOP has renal effects under basal conditions. Fourth, the study focused on acute injury at 24 h after reperfusion and did not assess long-term outcomes such as fibrosis, maladaptive repair, or progression from AKI to chronic kidney disease. Fifth, EX527 is a pharmacological SIRT1 inhibitor rather than a genetic knockout model, and off-target or context-dependent effects cannot be completely excluded. Finally, although SIRT1/PGC-1α signaling was implicated, downstream targets and alternative mechanisms, including inflammatory signaling, mitophagy, ferroptosis, and NAD+ metabolism, remain to be investigated.

## 5. Conclusions

In conclusion, SOP attenuated renal IRI-associated tubular injury, oxidative stress, apoptosis, and mitochondrial dysfunction in mice and reduced H/R-induced injury in HK-2 cells. These effects were associated with activation of the SIRT1/PGC-1α axis and were weakened by Sirt1 knockdown or pharmacological SIRT1 inhibition. SOP may therefore represent a candidate renoprotective compound for further preclinical investigation in ischemic kidney injury, although post-injury treatment models, long-term outcome studies, pharmacokinetic analyses, and clinical route optimization are required before translational conclusions can be drawn.

## Figures and Tables

**Table 1:** Sequence used for qPCR.

qPCR Primers			
Gene	Species	Sequences (Forward)	Sequences (Reverse)
SOD1	Human	5′-GACTGACTGAAGGCCTGCAT-3′	5′-ATCGGCCACACCATCTTTGT-3′
	Mouse	5′-GGAACCATCCACTTCGAGCA-3′	5′-TGATGGACGTGGAACCCATG-3′
SOD2	Human	5′-GGCCTACGTGAACAACCTGA-3′	5′-CCGTTAGGGCTGAGGTTTGT-3′
	Mouse	5′-AACTCAGGTCGCTCTTCAGC-3′	5′-CCTTGGACTCCCACAGACAC-3′
Catalase	Human	5′-AGTGATCGGGGGATTCCAGA-3′	5′-AAGTCTCGCCGCATCTTCAA-3′
	Mouse	5′-TCACTGACGAGATGGCACAC-3′	5′-ATCGAACGGCAATAGGGGTC-3′
Sirt1	Human	5′-TTACATTTTCCATGGCGCTGA-3′	5′-TGGCATGTCCCACTATCACT-3′
	Mouse	5′-TCAGAGTTGCCACCAACAC-3′	5′-TACTGGAACCAACAGCCTTA-3′
PGC-1α	Human	5′-TGAACAAGCACTTCGGTCA -3′	5′-CATCCATGGCTAGTCCTGA-3′
	Mouse	5′-ACTGAGCTACCCTTGGGATG-3′	5′-TAAGGATTTCGGTGGTGACA-3′
ACTB	Human	5′-CATGTACGTTGCTATCCAGGC-3′	5′-CTCCTTAATGTCACGCACGAT-3′
	Mouse	5′-GTGACGTTGACATCCGTAAAGA-3′	5′-GCCGGACTCATCGTACTCC-3′

**Table 2:** Antibodies used for Western blotting.

Antibodies			
Target	Dilution	Company	Catalog No.
**Catalase**	1:1000	Proteintech (Wuhan, China)	21260-1-AP
**DRP1**	1:2000	Proteintech (Wuhan, China)	12957-1-AP
**FIS1**	1:1000	Abclonal (Wuhan, China)	A5821
**MFN2**	1:5000	Proteintech (Wuhan, China)	12186-1-AP
**p-DRP1 (ser616)**	1:1000	CST (Shanghai, China)	4494T
**PGC-1α**	1:2000	Abclonal (Wuhan, China)	A12348
**Sirt1**	1:1000	Huabio (Hangzhou, China)	ER130811
**SOD1**	1:2000	Proteintech (Wuhan, China)	10269-1-AP
**SOD2**	1:2000	Proteintech (Wuhan, China)	24127-1-AP
**ACTB**	1:10,000	Proteintech (Wuhan, China)	66009-1-Ig

## Data Availability

Data are contained within the article. Additional raw data are available from the corresponding author upon reasonable request.

## Abbreviations

The following abbreviations are used in this manuscript:

IRI	ischemia–reperfusion injury
I/R	ischemia–reperfusion
AKI	acute kidney injury
SOP	sophocarpine
H/R	hypoxia–reoxygenation
MDA	malondialdehyde
SOD	superoxide dismutase
GSH	glutathione
ROS	reactive oxygen species
TRPA1	transient receptor potential anion channel 1
TRPV1	transient receptor potential vanilloid 1
BSA	bovine serum albumin
DAB	diaminobenzidine
TUNEL	TdT-mediated dUTP nick-end labeling
IF	immunofluorescence
MMP	mitochondrial membrane potential
ACTB	β-actin
DRP1	dynamin-related protein 1
SIRT1	sirtuin 1
PGC-1α	peroxisome proliferator-activated receptor gamma coactivator 1-alpha
NGAL	neutrophil gelatinase-associated lipocalin
TEM	transmission electron microscopy
DHE	dihydroethidium
RT-qPCR	real-time quantitative polymerase chain reaction
WB	Western blotting
MFN2	mitofusin 2
FIS1	mitochondrial fission 1 protein
